# Comprehensive Analysis of miRNome Alterations in Response to Sorafenib Treatment in Colorectal Cancer Cells

**DOI:** 10.3390/ijms17122011

**Published:** 2016-12-01

**Authors:** Anna-Maria Pehserl, Anna Lena Ress, Stefanie Stanzer, Margit Resel, Michael Karbiener, Elke Stadelmeyer, Verena Stiegelbauer, Armin Gerger, Christian Mayr, Marcel Scheideler, Georg C. Hutterer, Thomas Bauernhofer, Tobias Kiesslich, Martin Pichler

**Affiliations:** 1 Division of Oncology, Department of Internal Medicine, Medical University of Graz, 8010 Graz, Austria; anna@pehserl.com (A.-M.P.); anna.lena.ress@gmail.com (A.L.R.); stefanie.stanzer@medunigraz.at (S.S.); margit.resel@medunigraz.at (M.R.); verena.stiegelbauer@medunigraz.at (V.S.); armin.gerger@medunigraz.at (A.G.); thomas.bauernhofer@medunigraz.at (T.B.); 2 Research Unit of Non-Coding RNA and Genome Editing in Cancer, Medical University of Graz, 8010 Graz, Austria; 3 Department of Phoniatrics, ENT University Hospital, Medical University, 8010 Graz, Austria; michael.karbiener@medunigraz.at; 4 Institute of Pathology, Medical University of Graz, 8010 Graz, Austria; elke.stadelmeyer@medunigraz.at; 5 Laboratory for Tumour Biology and Experimental Therapies (TREAT), Institute of Physiology and Pathophysiology, Paracelsus Medical University, 5020 Salzburg, Austria; ch.mayr@salk.at (C.M.); t.kiesslich@salk.at (T.K.); 6 Department of Internal Medicine I, Salzburger Landeskliniken, Paracelsus Medical University, 5020 Salzburg, Austria; 7 Institute for Diabetes and Cancer (IDC), Helmholtz Zentrum München, German Research Center for Environmental Health, 85764 Neuherberg, Germany; marcel.scheideler@helmholtz-muenchen.de; 8 Joint Heidelberg-IDC Translational Diabetes Program, Heidelberg University Hospital, 69120 Heidelberg, Germany; 9 Molecular Metabolic Control, Medical Faculty, Technical University Munich, 85764 Munich, Germany; 10 German Center for Diabetes Research (DZD), 85764 Neuherberg, Germany; 11 Department of Urology, Medical University of Graz, 8010 Graz, Austria; georg.hutterer@medunigraz.at; 12 Department of Experimental Therapeutics, The University of Texas MD Anderson Cancer Center, Houston, TX 77054, USA

**Keywords:** colorectal cancer, sorafenib, miRNA

## Abstract

MicroRNAs (miRNAs) are master regulators of drug resistance and have been previously proposed as potential biomarkers for the prediction of therapeutic response in colorectal cancer (CRC). Sorafenib, a multi-kinase inhibitor which has been approved for the treatment of liver, renal and thyroid cancer, is currently being studied as a monotherapy in selected molecular subtypes or in combination with other drugs in metastatic CRC. In this study, we explored sorafenib-induced cellular effects in Kirsten rat sarcoma viral oncogene homolog olog (KRAS) wild-type and KRAS-mutated CRC cell lines (Caco-2 and HRT-18), and finally profiled expression changes of specific miRNAs within the miRNome (>1000 human miRNAs) after exposure to sorafenib. Overall, sorafenib induced a time- and dose-dependent growth-inhibitory effect through S-phase cell cycle arrest in KRAS wild-type and KRAS-mutated CRC cells. In HRT-18 cells, two human miRNAs (hsa-miR-597 and hsa-miR-720) and two small RNAs (SNORD 13 and hsa-miR-3182) were identified as specifically sorafenib-induced. In Caco-2 cells, nine human miRNAs (hsa-miR-3142, hsa-miR-20a, hsa-miR-4301, hsa-miR-1290, hsa-miR-4286, hsa-miR-3182, hsa-miR-3142, hsa-miR-1246 and hsa-miR-720) were identified to be differentially regulated post sorafenib treatment. In conclusion, we confirmed sorafenib as a potential anti-neoplastic treatment strategy for CRC cells by demonstrating a growth-inhibitory and cell cycle–arresting effect of this drug. Changes in the miRNome indicate that some specific miRNAs might be relevant as indicators for sorafenib response, drug resistance and potential targets for combinatorial miRNA-based drug strategies.

## 1. Introduction

Colorectal cancer (CRC) is among the leading causes of cancer-related mortality worldwide, thus representing a major public health problem [1]. It is the third most frequently diagnosed cancer, comprising 44% of all cancer cases in men [2]. CRC frequently arises from dysplastic adenomatous polyps, and the process of transformation to carcinoma involves several essential events [3]. These are characterized by the activation of oncogenes such as KRAS (Kirsten rat sarcoma viral oncogene homolog), c-MYC (v-myc avian myelocytomatosis viral oncogene homolog), and NRAS (neuroblastoma RAS viral oncogene homolog), and by inactivation of tumor suppressor genes such as APC (adenomatous polyposis coli) or DNA repair genes [4].

MicroRNAs (miRNAs) are non-coding, endogenous, single-stranded RNAs of ~22 nucleotides in length that play a role as post-transcriptional gene expression regulators by suppressing translation or inducing mRNA degradation of their target transcripts [5]. Many of them have been shown to be involved in essential biological and cellular processes such as inflammation, cell cycle regulation, stress response, differentiation, apoptosis and migration [6,7]. Moreover, several of them have been linked to colorectal carcinogenesis, prognosis and drug resistance in CRC patients [8,9].

Cytotoxic chemotherapy is the backbone of CRC treatment in clinical practice and improves survival rates in patients with metastatic CRC, especially when combined with targeted anti-angiogenic or anti-epidermal growth factor receptor (EGFR)-directed agents [10,11]. RAS mutations (KRAS and NRAS) have been associated with resistance against EGFR inhibitors and the testing for these genetic alterations is a common standard of care in CRC patients [12]. Sorafenib (Nexavar^®^) is a multi-tyrosine kinase inhibitor that blocks cell proliferation by inhibiting the mitogen-activated protein kinase (MAPK) pathway and prevents tumor-associated angiogenesis by inhibiting vascular endothelial growth factor receptors [13]. Based on large clinical trial data, sorafenib has been approved for the treatment of metastatic hepatocellular carcinoma (HCC), renal cell carcinoma and thyroid cancer [14,15,16].

Early pre-clinical and clinical data suggested a potential for this agent in combination with the standard chemotherapeutic drugs oxaliplatin and irinotecan in metastatic CRC patients [17,18]. However, the combination of sorafenib with standard FOLFOX (combination of 5-fluorocil, leucovorin and oxaliplatin) chemotherapy was not proven to be successful in terms of efficacy in a large phase II clinical trial using unselected first-line-treatment patients [19]. Nevertheless, further characterization of the molecular pharmacological effects of sorafenib in CRC [20] as well as elucidation of the therapeutic potential of sorafenib in particular molecular and genetic subtypes of metastatic CRC are currently ongoing [21,22].

Research has shown that miRNAs carry potential as therapeutic targets and the first clinical trials of miRNA-based drugs are underway [23]. Prior studies have found that miRNAs are involved in sorafenib resistance in hepatocellular carcinoma [24], and a pharmacological approach to manipulate those particular miRNAs was shown to overcome resistance in pre-clinical models and proposed combinatorial treatment options [13,25].

In this study, therefore, we investigated alterations in the miRNome in CRC cells following treatment with the anti-cancer drug sorafenib in order to describe for the first time in a descriptive manner the miRNA changes in CRC cell lines after sorafenib exposure.

## 2. Results

**Figure 1:**
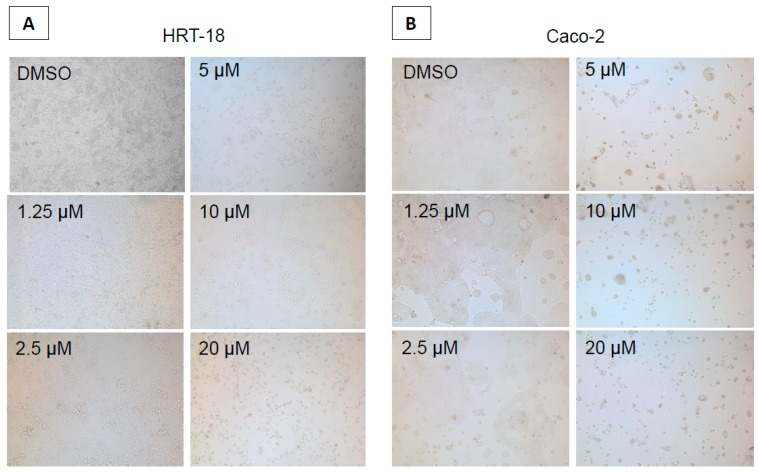
Sorafenib treatment for 72 h led to a dose-dependent decrease of viable cells in both cell lines. (**A**) The KRAS-mutated HRT-18 cell line and (**B**) The KRAS wild-type Caco-2 cell line (400× magnification).

**Figure 2:**
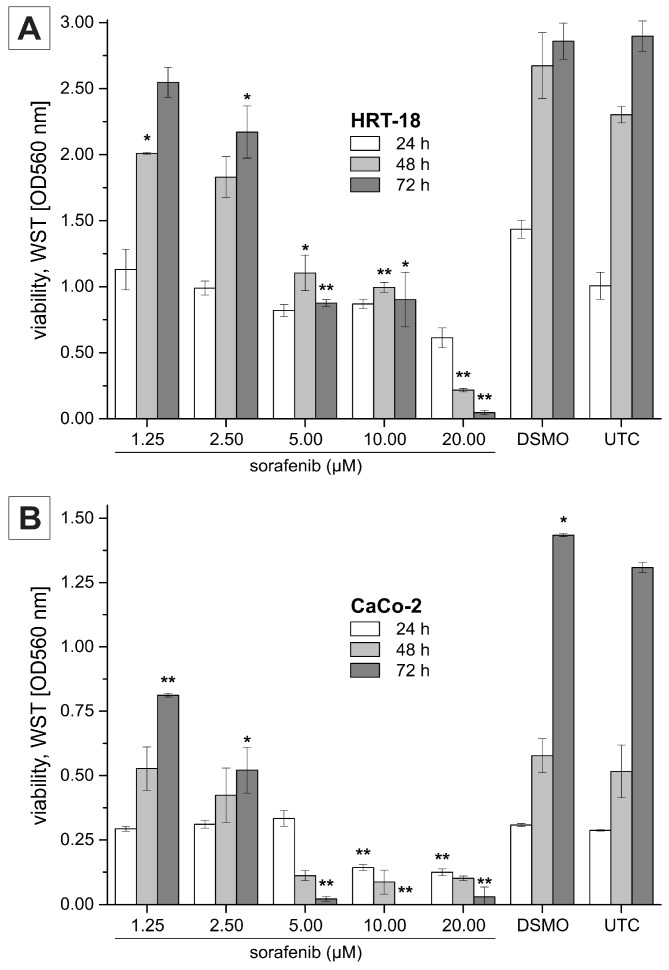
Dose- and time-dependent viability of (**A**) HRT-18 cells and (**B**) Caco-2 cells. Cells were seeded in 96-well microplates and treated with 1.25, 2.50, 5.00, 10.00 and 20.00 µM sorafenib. Viability was analyzed using WST-1 assay after 24, 48 and 72 h sorafenib treatment; data points represent mean values ± SEM of two independent experiments. ‘DMSO’ indicates samples treated with the solvent only (highest concentration). *, ** indicate values significantly (*p* < 0.05) or highly significantly (*p* < 0.01) different from the untreated control (UTC), respectively (two-sided, unpaired *t*-test).

**Figure 3:**
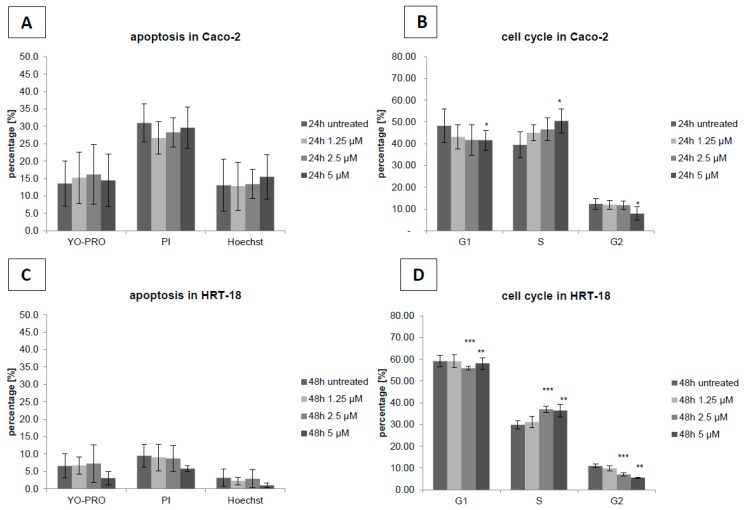
(**A**,**C**) No differences in staining patterns of YO-PRO, Hoechst and propidium iodide (PI) were observed after 24 h of sorafenib treatment in Caco-2 and after 48 h in HRT-18 cells and (**B**,**D**) A shift towards the S-phase in cell cycle was observed with increasing concentrations of sorafenib for both cell lines. *, **, *** indicate values significantly (*p* < 0.05), highly significantly (*p* < 0.01) or (*p* < 0.001) different from the solvent control, respectively (two-sided, unpaired *t*-test); untreated DMSO control indicates untreated cell samples.

**Figure 4:**
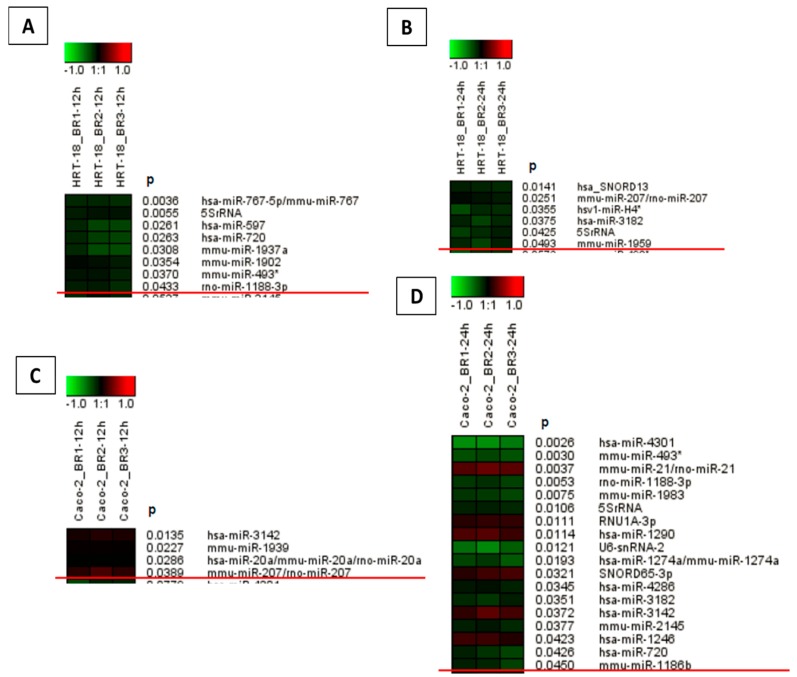
Microarray analysis identified several significantly differentially expressed miRNAs in the cell lines HRT-18 (**A**,**B**) and Caco-2 (**C**,**D**) upon sorafenib treatment compared to untreated controls (*p* < 0.05, red line indicated the border for significantly differential expressed miRNAs). Non-coding small RNAs were ranked based on differential expression. (**A**) HRT-18 cell expression analysis after 12 h (*n* = 3); (**B**) HRT-18 cell expression analysis after 24 h (*n* = 3); (**C**) Caco-2 cell expression analysis after 12 h (*n* = 3); and (**D**) Caco-2 cell expression analysis after 24 h (*n* = 3).

**Figure 5:**
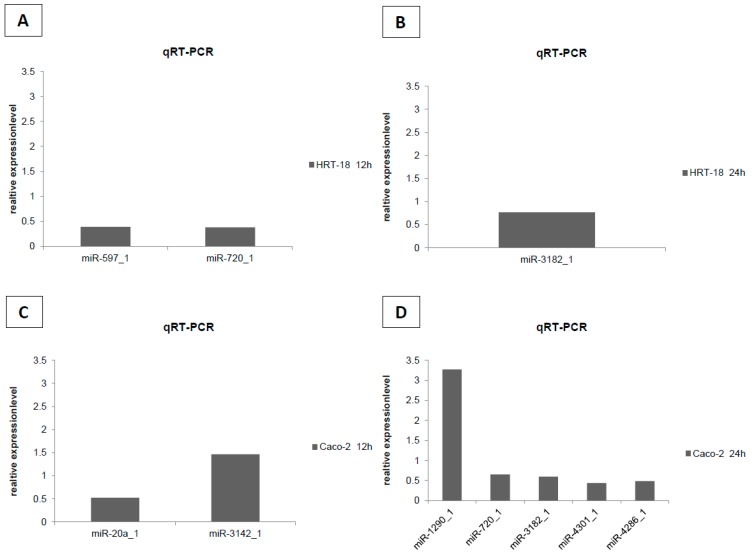
Quantitative RT-PCR (RT-qPCR) confirmation study of nine miRNAs. Except miR-767-3p, which was not detectable by RT-qPCR, we could confirm all other miRNAs detected by the microarray (miR-597, miR-720, miR-3182, miR-20a, miR-4301, miR-3142, miR-4286, miR-3142, miR-4286 and miR-1290). miRNA expressions normalized to SNORD68 are shown in relation to the untreated DMSO control. Representative examples of technical replicates are shown. (**A**) HRT-18 cell expression analysis after 12 h; (**B**) HRT-18 cell expression analysis after 24 h; (**C**) Caco-2 cell expression analysis after 12 h; and (**D**) Caco-2 cell expression analysis after 24 h.

To evaluate the effects of sorafenib on cellular growth in CRC cells, we used two independent screening methods for two different CRC cell lines (HRT-18 and Caco-2 cells). Both cell lines were treated with five different sequentially increasing concentrations of sorafenib (Dimethylsulfoxid (DMSO)-only control, 1.25, 2.5, 5, 10 and 20 µM) for 72 h. Overall, sorafenib significantly decreased the number of visible cells after 72 h in a dose-dependent manner in both cell lines after microscopic examination (Figure 1A,B). To obtain a more comprehensive time- and dose-dependent picture of the growth-inhibitory effects of sorafenib in both cell lines, we monitored cellular growth after 24, 48 and 72 h by the WST-1 assay. In line with the microscopic observations, both cell lines showed a significant dose- and time-dependent decrease in cellular growth (Figure 2A,B). Obviously, the KRAS wild-type Caco-2 cell line was more sensitive to sorafenib than the KRAS-mutated HRT-18 cells. Next we investigated the mode of action and whether sorafenib could induce apoptosis in the HRT-18 and Caco-2 cells. Using a multi-dye apoptosis assay, we did not get any significant differences in staining patterns (apoptotic activation) of YO-PRO, Hoechst and PI after 24 or 48 h of sorafenib exposure (Figure 3A,C). To investigate the role of sorafenib on cellular proliferation, we also analyzed the effect on cell cycle distribution in both cell lines by FACS after PI staining. Figure 3B,D show a significant decrease of cells in the G1-phase and an increase of cells arrested in the S-phase, depending on increasing concentrations of sorafenib. After confirming the inhibitory effect on cellular growth and selecting 5 µM sorafenib for further exposure (which is in the range of the sorafenib plasma concentration in patients) [26], we initiated a comprehensive microarray analysis to identify differentially expressed miRNAs in human colon cancer cells following exposure to sorafenib. To analyze a possible time-dependent course of miRNA alterations, we monitored the miRNome after 12 and 24 h in both cell lines. These relatively early time points were selected for miRNA profiling following the rationale that sorafenib is quickly inhibiting the signal transduction cascade, thereby leading to possible early miRNA expression changes. As the phenotype (cell cycle arrest) is changing over time, we did not measure the miRNA changes at later time points to avoid possible phenotype-associated indirect miRNA expression changes. For detection of differentially expressed miRNAs between sorafenib-treated and untreated cells, we used a comprehensive miRNA detection system which detects more than 1000 human miRNAs [27]. Heat maps for the comparison of miRNA expression levels after 12 and 24 h for both cell lines are shown in Figure 4. In HRT-18 cells, we identified four human miRNAs (hsa-miR-767, hsa-miR-597, and hsa-miR-720, hsa-miR-3182) and two human RNAs (SNORD13 and 5S rRNA) to significantly decrease in abundance after 12 and 24 h of sorafenib exposure, respectively (*p* < 0.05, Figure 4A,B). In the Caco-2 cell line, we identified two human miRNAs (hsa-miR-3142 and hsa-miR-20a) and eight human miRNAs (hsa-miR-4301, hsa-miR-1290, hsa-miR-4286, hsa-miR-3182, hsa-miR-3142, hsa-miR-1246 and hsa-miR-720) after 12 and 24 h, respectively (*p* < 0.05, Figure 4C,D). To validate the results of the microarray, we performed quantitative RT-PCR (RT-qPCR) for nine selected miRNAs using the same RNA extracted from sorafenib-treated and untreated control cells. The nine miRNAs were miR-767-3p, miR-597, miR-720, miR-3182, miR-20a, miR-4301, miR-3142, miR-4286 and miR-1290. With the exception of miR-767-3p (which was not detectable by RT-qPCR), we could confirm all other miRNAs detected by the microarray (Figure 5A–D).

## 3. Discussion

miRNAs play an important role in human molecular carcinogenesis and have been increasingly recognized as key regulators in many biological systems. In CRC they have potential as biomarkers for a patient’s prognosis, prediction of treatment response and drug targets [9]. In our study, it was reasonable to hypothesize that the anti-neoplastic drug sorafenib could lead to changes in miRNA gene expression, since it inhibits the maturation and proliferation of malignant cells [13]. First, we established sorafenib as a growth-inhibitory factor in two independent cell lines. The observation that the KRAS wild-type cell line Caco-2 is more sensitive to sorafenib than the KRAS-mutated HRT-18 cell line is interesting, but also in line with previous experimental studies which showed efficacy for sorafenib in both KRAS wild-type/-mutated subtypes. Interestingly, in this context, a clinical trial showed a longer overall survival in KRAS wild-type patients treated with regorafenib (a “sister” agent of sorafenib) compared to those with KRAS-mutated tumors [21].

Second, we observed a growth-inhibitory effect and an arrest in the S-phase of the cell cycle as the mode of the cytotoxic action of sorafenib, which has been previously described in papillary thyroid carcinoma cell lines (BHT101, B-CPAP and TK1) and anaplastic cell lines (SW1736 and Hth7) [28]. Furthermore, in medulloblastoma, the effect of sorafenib in Daoy cells showed a decrease of cells in the G1-phase and an increase of cells arrested in the S-phase [29]. In our study, using microarray analyses in two different cell lines, we identified and confirmed two differentially expressed human miRNAs (hsa-miR-597 and hsa-miR-720) in HRT-18 and identified nine differentially expressed human miRNAs (hsa-miR-3142, hsa-miR-20a, hsa-miR-4301, hsa-miR-1290, hsa-miR-4286, hsa-miR-3182, hsa-miR-3142, hsa-miR-1246 and hsa-miR-720) in Caco-2 cells. Except for miR-3142 and miR-1290, whose expressions were up-regulated, all other miRNAs were down-regulated after treatment with sorafenib. For confirmation we performed RT-qPCR for the nine selected miRNAs. With the exception of miR-767-3p, which was not detectable by RT-qPCR, we could confirm the regulation of all the other miRNAs detected by the microarray (miR-597, miR-720, miR-3182, miR-20a, miR-4301, miR-3142, miR-4286 and miR-1290). Most of them are miRNAs with previously reported roles in cancer and some show similar changes in expression patterns following photodynamic anticancer treatment in human A31 cells [30]. The expression status of some of these miRNAs in cancer is correlated with clinical outcome and some of their target genes are recognized as tumor suppressors. For instance, miR-720 has been shown to be significantly up-regulated in CRC tumor tissue compared to non-cancer samples [31]. High expression of miR-720 was also significantly associated with male gender, lymph node metastases [32], tumor size, and distant metastasis, which led to a poor five-year overall survival rate in CRC patients. In colorectal carcinogenesis, miR-720 acts as a promoting factor and could serve as a prognostic indicator in the development of CRC [31].

Another example is miR-20a, which plays a key role in tumorigenesis and progression and is involved in the carcinogenesis of gastric cancer through modulation of the EGFR2 signaling pathway [33]. The high endogenous expression level represents an attractive, therapeutic vulnerability and makes miR-20a a target for miRNA inhibitors in patients with CRC [34]. It acts as a tumor suppressor in thyroid cancer cells and targets LIMK1 [35,36]. Furthermore, miR-20a expression has been related to the malignant process of cervical cancer, especially invasion and metastasis by targeting ATG7 and TIMP2 [37].

Higher expression levels of miR-1290 were positively correlated with high tumor stage and positive lymph node metastasis in non-small cell lung cancer (NSCLC). This indicates that miR-1290 is up-regulated in NSCLC and may be used as a potential prognostic biomarker [38]. Additionally, miR-1290 is also involved in LSCC (laryngeal squamous cell carcinoma) pathogenesis and cervical cancer [39,40]. Down-regulation of miR-597 has been identified in CRCs and other malignancies [41]. No relevant reports about the relationship between miR-3182, miR-4301, miR-3142, miR-4286 and cancer have been found.

In general, microRNAs have been already proposed as novel predictive and prognostic biomarkers in CRC [42]. For instance, miR-31-3p has been involved in the survival of KRAS wild-type patients treated with an EGFR inhibitor [43]. Interestingly, microRNA-31 has been shown to activate RAS signaling by repressing RAS p21 GTPase activating protein 1 [44]. More evidence for the involvement of miR-31-3p in RAS signaling and EGFR inhibitor resistance came from a number of other studies, which make this microRNA a potential biomarker for future evaluation in prospective clinical trials [45,46,47,48]. Though we did not detect increased apoptosis activation in our cell models, there have been some reports about the pro-apoptotic effect of sorafenib in liver cancer in in vivo models [49].

In conclusion, our study confirms the growth-inhibitory effects of sorafenib in CRC cells through S-phase cell cycle arrest. For the first time, we analyzed the alterations of the miRNome after sorafenib exposure. The miRNA candidates identified in this study might be interesting resistance factors or biomarkers for sorafenib sensitivity, and further testing in clinical samples of sorafenib-treated CRC patients is warranted.

## 4. Material and Methods

### 4.1. Colorectal Cancer Cell Lines

The CRC cell lines Caco-2 (KRAS wild-type) and HRT-18 (KRAS-mutated) were purchased from American Type Culture Collection. Caco-2 was cultured in MEM with Earle’s salts (PAA Laboratories, Pasching, Austria) supplemented with 2 mmol/L of l-glutamine, 1.5 g/L of sodium bicarbonate, 0.1 mmol/L of nonessential amino acids, 50 units/mL of penicillin, 50 µg/mL of streptomycin, and 10% FBS (PAA Laboratories). HRT-18 cells were maintained in RPMI 1640 (GIBCO, Vienna, Austria) containing 2 mmol/L of l-glutamine, 50 units/mL of penicillin, 50 µg/mL of streptomycin, and 10% FBS. After obtaining a confluence of approximately 80%, total RNA was isolated following a standard TRIzol protocol and RNA was stored at −70 °C until further procedures.

### 4.2. Cytotoxicity Assays

Cancer cells were seeded at a density of 2–5 × 10^3^ cells/well in a 96-well microtiter plate and were allowed to attach overnight. Freshly prepared sorafenib (BioVisions Inc., Milpitas, CA, USA) was then added with the final concentration ranging from 0 to 20 µM. After 24, 48 and 72 h, cell viability was assessed by light microscopy and using a WST-1 assay (Roche, Mannheim, Germany) as previously described [50]. Three independent experiments were performed in quadruplicate, and the data reported represent the mean ± SD.

### 4.3. Cell Cycle and Apoptosis

For analyses of cell cycle and apoptosis, Caco-2 and HRT-18 cells were detached from the culture dishes with Accutase (Gibco, Thermo Fischer Scientific, Waltham, MA, USA). After 20 min at room temperature incubation of the dissolved cells with a hypotonic lysis solution containing 50 µg/mL propidium iodide (PI; Sigma, Vienna, Austria) and 100 µg/mL RNAse (Fermentas, Thermo Fischer Scientific), cells were stored on ice in the dark for maximum one hour until flow cytometric measurement were performed. The analysis of apoptosis was done with the Chromatin Condensation/Membrane Permeability/Dead Cell Apoptosis Kit with Hoechst 33342/YO-PRO^®^-1 and PI for Flow Cytometry (Thermo Fischer Scientific) which was performed according to the manufacturer’s instructions. The YO-PRO^®^-1 signal indicates apoptotic cells, the propidium iodide signal dead cells and bright Hoechst staining condensed chromatin of apoptotic cells.

Side scatter and forward scatter profiles were used to eliminate cell doublets. The samples were acquired on a LSRII flow cytometer (BD Bioscience, Schwechat, Austria) and data were analyzed with the DIVA software (BD Bioscience) or for analyses of the cell cycle with the ModFit LT software (Verity Software House, Topsham, ME, USA).

### 4.4. MicroRNA Microarray Analysis

Microarrays were generated by spotting Nexterion HiSens E Slides (Schott, ordered by Peqlab, Prod.no. 39-1125813, Erlangen, Germany) with the miRCURY LNA™ microRNA Array ready-to-spot probe set, sixth gen, human, mouse and rat (Exiqon, Vebeak, Denmark). This probe set consisted of 2383 unique capture probes, covering all mature miRNAs from human, mouse and rat annotated in miRBase version 16.0. For two-color hybridizations, 1 µg total RNA of each sample was used for Hy3 and Hy5 labeling using the miRCURY LNA™ microRNA Hi-Power Labeling Kit (Exiqon) according to the manufacturer’s instructions and as published previously [51]. Hybridizations were performed on a TECAN HS400 Hybridization Station (Tecan, Männedorf, Switzerland). All hybridizations were repeated with reversed dye assignment (dye-swap). Hybridized slides were scanned with a GenePix 4000B microarray scanner (Molecular Devices, Sunnyvale, CA, USA) at 10 µm resolution and the resultant TIFF images were analyzed with GenePix Pro 4.1 software. Raw data was subsequently processed using ArrayNorm for normalization [52] and Genesis software for evaluation [53]. Up- or down-regulation of individual miRNAs was tested by employing a one-sample *t*-test using the log2-transformed expression ratios (i.e., log2-fold changes regarding sorafenib-treated versus no treatment). The analysis was based on miRNA-level expression data. It should however be noted that in the array platform chosen by us (Exiqon LNA-based oligonucleotide probes), each miRNA is detected by a single probe (spotted in eight technical replicates on one chip). miRNAs with a corresponding *p*-value < 0.05 were considered as statistically significant.

### 4.5. Validation of Microarray by RT-qPCR

As previously published [30], the same RNA samples harvested for microarray analyses were subsequently used for confirmation by quantitative RT-PCR. cDNA was synthesized from 500 ng of total RNA using miScript Reverse Transcription Kit (Qiagen, Hilden, Germany). Quantification of miRNAs was performed using the miScript SYBR Green PCR kit (Qiagen, Hilden, Germany) and the Hs_miScript Primer Assays (Hs_miR-3142_1 miScript Primer Assay (100), Hs_miR-767-3p_1 miScript Primer Assay (100), Hs_miR-597_1 miScript Primer Assay (100), Hs_miR-720_1 miScript Primer Assay (100), Hs_miR-3182_1 miScript Primer Assay (100), Hs_miR-20a_1 miScript Primer Assay (100), Hs_miR-4301_1 miScript Primer Assay (100), Hs_miR-1290_1 miScript Primer Assay (100), Hs_miR-4286_1 miScript Primer Assay (100), Hs_miR-3182_1 miScript Primer Assay (100), Hs_miR-1246_1 miScript Primer Precursor Assay (100) and Hs_SNORD68_1 miScript Primer Assay (100), Qiagen) according to the manufacturer’s recommendations on a Light Cycler 480 real-time PCR device (Roche, Mannheim, Germany). RT-qPCR was carried out using normalization to SNORD68 as published previously [53].
